# Probing Serum Albumins and Cyclodextrins as Binders of the Mycotoxin Metabolites Alternariol-3-Glucoside, Alternariol-9-Monomethylether-3-Glucoside, and Zearalenone-14-Glucuronide

**DOI:** 10.3390/metabo13030446

**Published:** 2023-03-18

**Authors:** Miklós Poór, Beáta Lemli, Péter Vilmányi, Ágnes Dombi, Zoltán Nagymihály, Eszter Borbála Both, Nándor Lambert, Tamás Czömpöly, Lajos Szente

**Affiliations:** 1 Department of Pharmacology, Faculty of Pharmacy, University of Pécs, Rókus u. 2, H-7624 Pécs, Hungary; 2 Food Biotechnology Research Group, János Szentágothai Research Centre, University of Pécs, Ifjúság útja 20, H-7624 Pécs, Hungary; 3 Green Chemistry Research Group, János Szentágothai Research Centre, University of Pécs, Ifjúság útja 20, H-7624 Pécs, Hungary; 4 Soft Flow Ltd., Pellérdi út 91/B, H-7634 Pécs, Hungary; 5 CycloLab Cyclodextrin Research & Development Laboratory, Ltd., H-1097 Budapest, Hungary

**Keywords:** alternariol glucosides, zearalenone glucuronide, serum albumins, cyclodextrins, mycotoxin binders

## Abstract

Mycotoxins are toxic metabolites of molds. Chronic exposure to alternariol, zearalenone, and their metabolites may cause the development of endocrine-disrupting and carcinogenic effects. Alternariol-3-glucoside (AG) and alternariol-9-monomethylether-3-glucoside (AMG) are masked derivatives of alternariol. Furthermore, in mammals, zearalenone-14-glucuronide (Z14Glr) is one of the most dominant metabolites of zearalenone. In this study, we examined serum albumins and cyclodextrins (CDs) as potential binders of AG, AMG, and Z14Glr. The most important results/conclusions were as follows: AG and AMG formed moderately strong complexes with human, bovine, porcine, and rat albumins. Rat albumin bound Z14Glr approximately 4.5-fold stronger than human albumin. AG–albumin and Z14Glr–albumin interactions were barely influenced by the environmental pH, while the formation of AMG–albumin complexes was strongly favored by alkaline conditions. Among the mycotoxin–CD complexes examined, AMG–sugammadex interaction proved to be the most stable. CD bead polymers decreased the mycotoxin content of aqueous solutions, with moderate removal of AG and AMG, while weak extraction of Z14Glr was observed. In conclusion, rat albumin is a relatively strong binder of Z14Glr, and albumin can form highly stable complexes with AMG at pH 8.5. Therefore, albumins can be considered as affinity proteins with regard to the latter mycotoxin metabolites.

## 1. Introduction

Mycotoxins are toxic secondary metabolites of filamentous fungi. Their frequent occurrence in food and animal feed is a significant economic risk and health threat. Alternariol is produced by *Alternaria* species [1], while zearalenone is typically formed by *Fusarium* fungi [2]. Based on previous studies, chronic exposure to alternariol, zearalenone, and/or some of their metabolites may lead to the development of endocrine-disrupting and carcinogenic effects [3,4].

**Figure 1:**
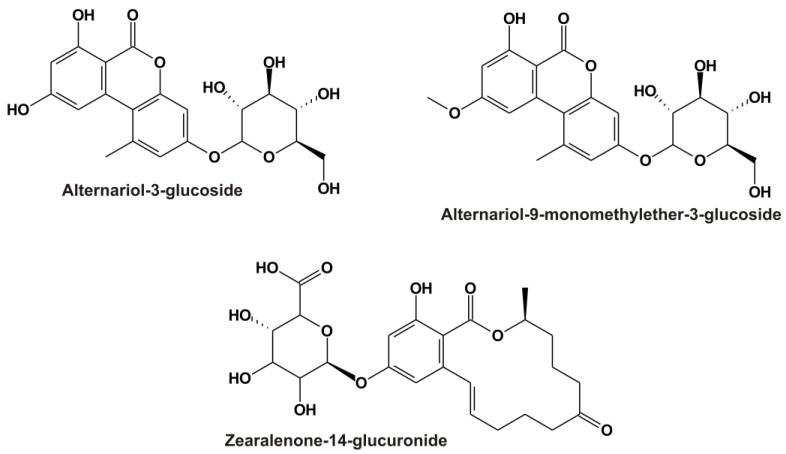
Chemical structures of alternariol-3-glucoside (AG), alternariol-9-monomethylether-3-glucoside (AMG), and zearalenone-14-glucuronide (Z14Glr).

*Alternaria* toxins are common contaminants in grains, sunflower seeds, tomatoes, apples, and their corresponding products (e.g., fruit and vegetable juices, wine, and beer) [1,5]. In addition to the parent *Alternaria* toxins, their modified derivatives also appear in certain food products, including the masked mycotoxins alternariol-3-glucoside (AG; Figure 1) and alternariol-9-monomethylether-3-glucoside (AMG; Figure 1) [6,7]. Masked metabolites are typically less toxic; however, they can be hydrolyzed in mammals, leading to the release of the parent mycotoxins [1,8].

Zearalenone and its derivatives commonly contaminate grains and cereal products (e.g., maize and maize-rich food) [4]. Phase I and II reactions take part in the biotransformation of zearalenone in mammals, where reduction and glucuronidation are the most important processes [2]. Glucuronic acid conjugates of zearalenone and zearalenols, such as zearalenone-14-glucuronide (Z14Glr; Figure 1), are dominant metabolites in blood, urine, and tissues [2].

Serum albumin is one of the most abundant plasma proteins in the circulation; it can form highly stable complexes with certain xenobiotics, affecting the toxicokinetic properties of the bound compounds [9]. Some mycotoxins bind to albumin with high affinity, including alternariol [10], zearalenone [11], and ochratoxin A [12]. In addition, albumin can be considered as an affinity protein in sample preparation and purification; it has been successfully applied for the extraction of alternariol [13] and ochratoxin A [14,15] from aqueous matrices. A recent study also demonstrated the strong interactions of methyl and sulfate metabolites of alternariol with serum albumins [16].

Cyclodextrins (CDs) are cyclic oligosaccharides containing glucose subunits. CDs can accommodate lipophilic ligand molecules in their apolar cavity, forming host–guest-type inclusion complexes [17]. As has been recently reported, alternariol and its methyl and sulfate metabolites form relatively stable complexes with sulfobutylether-β-CD (SBECD) and with sugammadex (SGD; a chemically modified γ-CD) [16,18]. Furthermore, SGD bound alternariol and its methyl derivative with unexpectedly high affinity [16,18]. Alternariol and its methyl/sulfate metabolites were successfully extracted from aqueous solutions with insoluble (but water-swellable) β-CD bead polymer (BBP) [16,19]. Moreover, BBP also strongly decreased the alternariol content of red wine [13]. Zearalenone, zearalenols, zearalanone, zearalanols, and zearalenone sulfate were almost completely removed from aqueous solutions by BBP [20,21,22]. In addition, certain CDs strongly alleviated or even ceased the alternariol- and zearalenone-induced toxic effects in cell experiments and in zebrafish studies [18,23]. These observations demonstrate that CDs are promising binders of these mycotoxins.

As mentioned in the previous two paragraphs, the interactions of serum albumins and CDs have been investigated with several metabolites of alternariol and zearalenone. However, the complex formation of AG, AMG, and Z14Glr with these host molecules has not yet been tested. Since the binding affinity of alternariol and zearalenone metabolites toward albumins and CDs commonly shows large variations [11,16], we felt it reasonable to test these host molecules as potential binders of AG, AMG, and Z14Glr. As highlighted by previous studies, albumin is suitable for the extraction of certain mycotoxins (e.g., alternariol and ochratoxin A) from aqueous solutions and from certain beverages [13,14,15]. Therefore, albumin can be a cheaper and widely available alternative of antibodies, which may have high importance with regard to emerging mycotoxins and masked/modified metabolites (where antibodies are typically not commercially available). The analytical extraction, purification, and quantification of modified mycotoxin derivatives are very challenging, due to the distinct physicochemical properties of these metabolites compared to their parent mycotoxins [24]. Insoluble CD polymers can effectively extract certain xenobiotics from aqueous solutions; therefore, they may be applied for the treatment of wastewater and/or for the removal of toxicants, pollutants, or pharmaceutical residues from aqueous matrices [25,26,27]. Based on previous studies, BBP is highly suitable for the extraction of alternariol, alternariol sulfate, alternariol methyl ether, alternariol monomethyl sulfate, zearalenone, zearalenols, zearalanone, zearalanols, zearalenone sulfate, and ochratoxin A [16,19,20,21,22]. Nevertheless, this polymer showed moderate (e.g., citrinin, sterigmatocystin, and zearalenone glucoside) or slight (e.g., deoxynivalenol and aflatoxin M1) interactions with certain other mycotoxins [21,22].

Considering the abovementioned data, in the present study, the interactions of AG, AMG, and Z14Glr were examined with human, bovine, porcine, and rat serum albumins, as well as with SBECD, SGD, and CD polymers. Our main goal was to identify potentially suitable mycotoxin binders that could be applied for toxin removal and/or analytical sample preparation. Furthermore, Z14Glr is a major circulating metabolite of zearalenone. Therefore, the characterization of its interaction with albumin can also be interesting from the toxicokinetic point of view.

## 2. Materials and Methods

### 2.1. Reagents

Alternariol-3-glucoside (AG) and alternariol-9-monomethylether-3-glucoside (AMG) were obtained from ASCA GmbH (Berlin, Germany). The synthesis, purification, and structural analysis of zearalenone-14-β,D-glucuronide (Z14Glr) are described in the Supplementary Materials. Alternariol was purchased from Cfm Oskar Tropitzsch GmbH (Marktredwitz, Germany). Zearalenone, human serum albumin (HSA), bovine serum albumin (BSA), porcine serum albumin (PSA), and rat serum albumin (RSA) were obtained from Merck (Darmstadt, Germany). Sulfobutylether-β-cyclodextrin (SBECD), sugammadex (SGD), insoluble β-cyclodextrin bead polymer (BBP; epichlorohydrin crosslinked bead polymer; BCD content: 50 m/m%), and γ-cyclodextrin bead polymer (GBP; epichlorohydrin cross-linked bead polymer; GCD content: 60 m/m%) were obtained from CycloLab Cyclodextrin Research and Development Laboratory, Ltd. (Budapest, Hungary). Mycotoxin stock solutions (10 mM; stored at −20 °C) were prepared in dimethyl sulfoxide.

### 2.2. Spectroscopic Studies

Fluorescence emission spectra were collected at 25 °C using a Hitachi F-4500 fluorescence spectrophotometer (Tokyo, Japan). The quenching effects of AG, AMG, and Z14Glr (each 0–5 μM) on the emission signals of albumins (2 μM each) were examined at 340 nm in phosphate-buffered saline (PBS, pH 7.4). UV–vis measurements were performed at 25 °C in PBS, employing a Jasco V730 UV–vis spectrophotometer (Tokyo, Japan). Thereafter, we corrected the inner-filter effects of the mycotoxins, as previously reported [11,28]. Stern–Volmer quenching constants (*K_SV_*; L/mol) were calculated with the graphical application of the Stern–Volmer equation [11,29]:

(1) $$\frac{I_{0}}{I}=1+K_{SV}\times \left[Q\right]$$

where *I*_0_ and *I* are the emission intensities of the protein without and with the quencher, respectively, while [*Q*] is the concentration (mol/L) of the quencher (mycotoxin). The binding constants (*K*; L/mol) of mycotoxin–albumin complexes were determined with nonlinear fitting, using the HyperQuad2006 software (version 3.1; Protonic Software GmbH, Hanau, Germany), as described previously [11,30].

The impacts of SBECD (0–10 mM) and SGD (0–5 mM) on the emission signals of mycotoxins (1 μM each) were tested in sodium acetate buffer (0.05 M, pH 5.0). Mycotoxin–CD interactions were evaluated using the Benesi–Hildebrand equation [11,31], assuming 1:1 stoichiometry of complex formation:

(2) $$\frac{F_{0}}{\left(F-F_{0}\right)}=\frac{1}{A}+\frac{1}{A\times K\times [CD]}$$

where *K* (L/mol) is the binding constant, *F*_0_ is the emission signal of the mycotoxin without the host molecules, *F* is the emission signal of the mycotoxin with CDs, *A* is a constant, and [*CD*] is the molar concentration (mol/L) of the host molecules.

### 2.3. Ultracentrifugation Studies

To confirm the data calculated based on fluorescence quenching studies, ultracentrifugation experiments were also performed. With the proper conditions of ultracentrifugation, albumin (with the bound ligands) can be sedimented [16,32]. The samples (final volume: 500 μL) contained AG, AMG, or Z14Glr (5 μM each) with HSA (60 or 180 μM) dissolved in PBS. Centrifugation (16 h, 170,000× *g*, 20 °C) was performed using 11 × 34 mm PC tubes (Beckman Coulter, Brea, CA, US) and a Beckman Coulter Optima MAX-XP tabletop ultracentrifuge (fixed-angle rotor). The unbound fractions of mycotoxins were determined in the protein-free supernatant by HPLC-FLD (see details in Section 2.5), as previously reported [32].

(3) $$K=\frac{\left[LA\right]}{\left[L\right]\times [A]}$$

where [*L*], [*A*], and [*LA*] are the concentrations (mol/L) of the ligand (unbound), albumin (unbound), and the ligand–albumin complex, respectively.

Furthermore, to test the impact of environmental pH on the stability of mycotoxin–albumin complexes, the abovementioned experiments were also performed in 0.05 M sodium acetate (pH 5.0) and 0.05 M sodium borate (pH 8.5) buffers.

### 2.4. Extraction of Mycotoxins with Cyclodextrin Bead Polymers

The extraction of mycotoxins by insoluble (but water-swellable) CD bead polymers was tested in 0.05 M sodium acetate (pH 5.0) and 0.05 M sodium borate (pH 10.0) buffers. AG, AMG, alternariol, Z14Glr, and zearalenone solutions (5 μM each) were incubated with increasing amounts (0–10 mg/mL) of BBP or GBP in a thermomixer for 40 min at 1000 rpm and 25 °C. After pulse centrifugation (5000× *g*, 3 s), the mycotoxin contents of the supernatants were analyzed by HPLC-FLD (see Section 2.5).

### 2.5. HPLC Analyses

The quantitative analyses of the examined mycotoxins were performed with a Jasco HPLC system (Tokyo, Japan) equipped with a binary pump (PU-4180), an autosampler (AS 4050), and a fluorescence detector (FP-920). Chromatograms were evaluated using the ChromNAV2 software (Jasco). Alternariol [19] and zearalenone [20] were analyzed as described previously. During the validation of our new HPLC-FLD methods, the linearity (LIN), limit of detection (LOD), limit of quantification (LOQ), and intraday repeatability (IDR) were determined as described previously [13].

AG and AMG were quantified using the following method: Isocratic elution of the samples (20 μL) was performed at room temperature with a 1 mL/min flow rate, employing phosphoric acid (1 mM) and acetonitrile (65:35 *v*/*v*%) as the mobile phase, where a Security Guard (C18, 4.0 × 3.0 mm; Phenomenex, Torrance, CA, US) precolumn was linked to a Kinetex EVO C18 (250 × 4.6 mm, 5 μm; Phenomenex) analytical column. Chromatograms were recorded, applying 335 nm and 455 nm excitation and emission wavelengths, respectively. The method validation parameters were as follows: AG (LIN: 0.2–5.0 μM, R^2^ = 0.999; LOD = 0.05 μM; LOQ = 0.18 μM; IDR = 1.3%, *n* = 7); AMG (LIN: 0.2–5.0 μM, R^2^ = 0.997; LOD = 0.15 μM; LOQ = 0.50 μM; IDR = 2.3%, *n* = 7).

Z14Glr was quantified using the following method: Isocratic elution of the samples (20 μL) was performed at room temperature with a 1 mL/min flow rate, employing 1% acetic acid solution and acetonitrile (65:35 *v*/*v*%) as the mobile phase, where a Security Guard (C8, 4.0 × 3.0 mm; Phenomenex) precolumn was linked to a Mediterranea See8 (C8, 150 × 4.6 mm, 5 μm; Teknokroma, Barcelona, Spain) analytical column. Chromatograms were recorded, applying 315 nm and 465 nm excitation and emission wavelengths, respectively. The method validation parameters for the HPLC analysis of Z14Glr were also determined (LIN: 0.2–5.0 μM, R^2^ = 0.999; LOD = 0.15 μM; LOQ = 0.50 μM; IDR = 2.0%, *n* = 7).

### 2.6. Statistical Analyses

Statistical significance (*p* < 0.01) was evaluated using one-way ANOVA (with Tukey’s post hoc test), employing the SPSS Statistics program (IBM, Armonk, NY, USA).

## 3. Results and Discussion

### 3.1. Interaction of AG, AMG, and Z14Glr with Serum Albumins Based on Fluorescence Quenching Studies

The interaction of a ligand molecule with albumin usually induces a decrease in the emission signal of the protein [33]; therefore, fluorescence quenching studies were performed. In PBS (pH 7.4), increasing amounts of AG, AMG, and Z14Glr were added to standard concentrations of human (HSA), bovine (BSA), porcine (PSA), or rat (RSA) serum albumins, after which the emission spectra were recorded.

**Figure 2:**
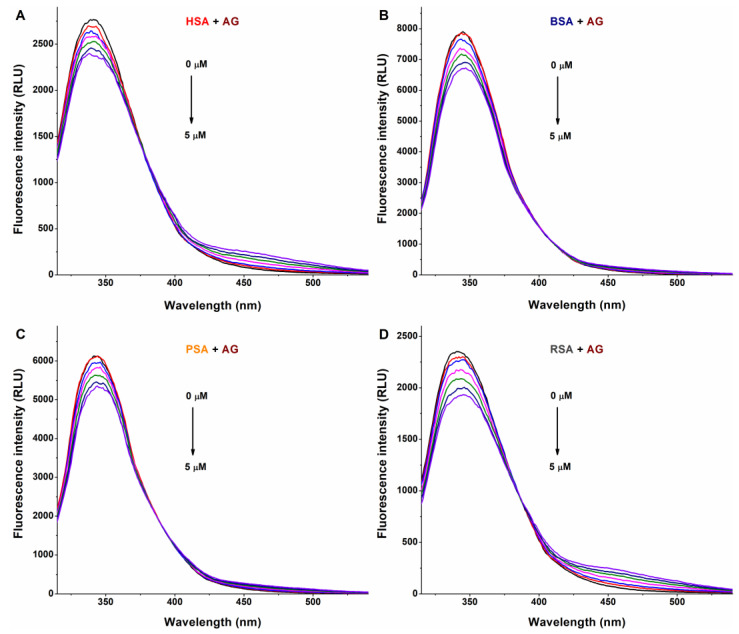
Representative fluorescence emission spectra of albumins, and the quenching effects of alternariol-3-glucoside (AG): Impacts of increasing concentrations of AG (0–5 μM) on the emission signals of human (HSA; (**A**)), bovine (BSA; (**B**)), porcine (PSA; (**C**)), and rat (RSA; (**D**)) serum albumins in PBS (pH 7.4; albumin concentration: 2 μM; λ_ex_ = 295 nm).

**Figure 3:**
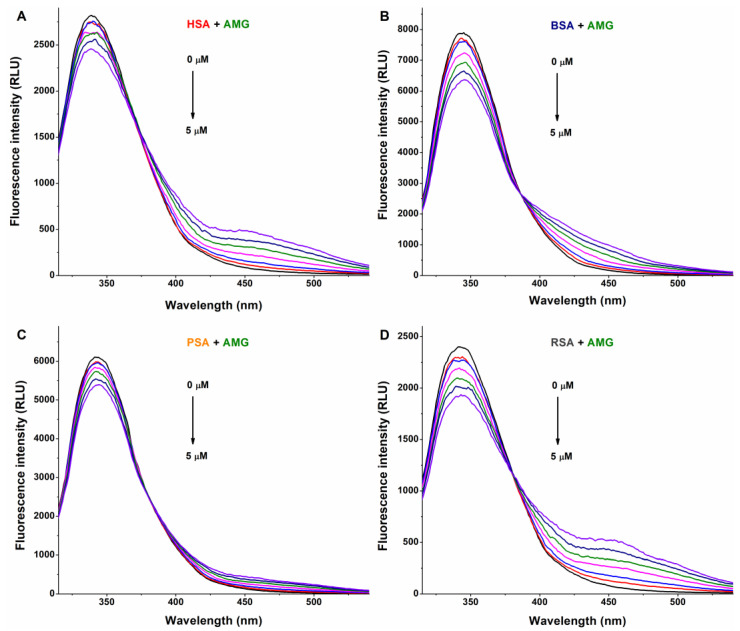
Representative fluorescence emission spectra of albumins, and the quenching effects of alternariol-9-monomethylether-3-glucoside (AMG): Impacts of AMG (0–5 μM) on the emission signals of human (HSA; (**A**)), bovine (BSA; (**B**)), porcine (PSA; (**C**)), and rat (RSA; (**D**)) serum albumins in PBS (pH 7.4; albumin concentration: 2 μM; λ_ex_ = 295 nm).

In a concentration-dependent fashion, AG (Figure 2) and AMG (Figure 3) induced a gradual decrease in the emission signal (around 340 nm) of each albumin tested. The inner-filter effects of mycotoxins were corrected; therefore, these observations demonstrate the formation of AG–albumin and AMG–albumin complexes. The appearance of a further, smaller peak at higher wavelengths resulted from the intrinsic fluorescence of alternariol glucosides, which did not affect the evaluation at 340 nm.

**Figure 4:**
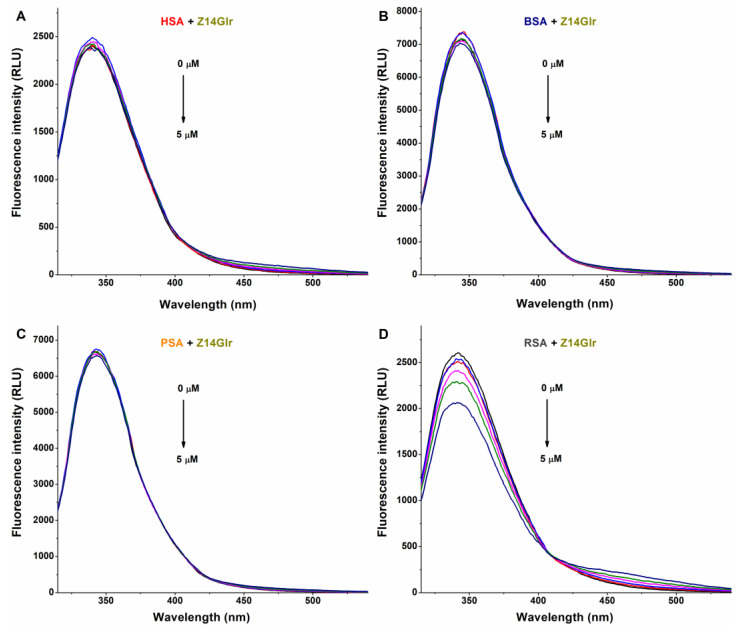
Representative fluorescence emission spectra of albumins, and the quenching effects of zearalenone-14-glucuronide (Z14Glr): Impacts of Z14Glr (0–5 μM) on the emission signals of human (HSA; (**A**)), bovine (BSA; (**B**)), porcine (PSA; (**C**)), and rat (RSA; (**D**)) serum albumins in PBS (pH 7.4; albumin concentration: 2 μM; λ_ex_ = 295 nm).

The fluorescence emission spectra of HSA, BSA, and PSA were barely affected by Z14Glr (Figure 4). However, the emission intensity of RSA was considerably decreased by Z14Glr, suggesting the interaction of the mycotoxin with rat albumin. 

**Figure 5:**
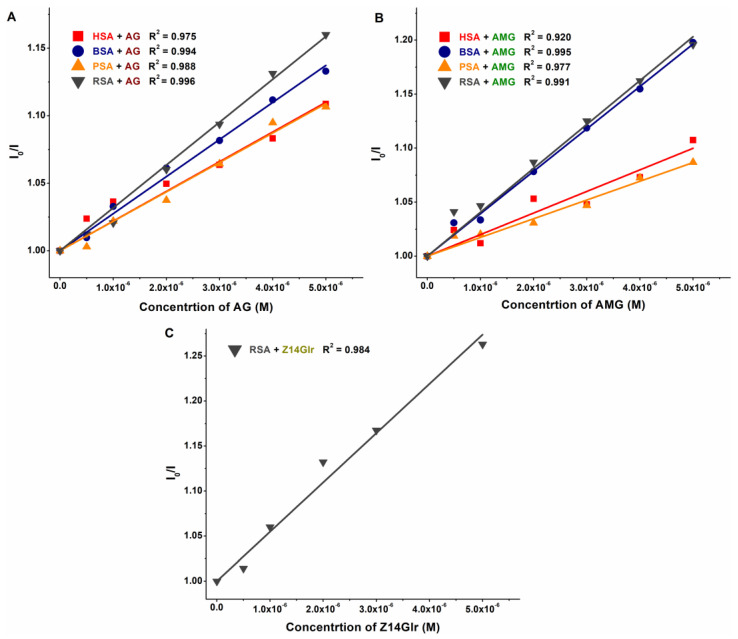
Stern–Volmer plots of AG–albumin (**A**), AMG–albumin (**B**), and Z14Glr–RSA (**C**) complexes (PBS, pH 7.4; λ_ex_ = 295 nm, λ_em_ = 340 nm; albumin concentration: 2 μM; *n* = 3; AG, alternariol-3-glucoside; AMG, alternariol-9-monomethylether-3-glucoside; Z14Glr, zearalenone-14-glucuronide; HSA, human serum albumin; BSA, bovine serum albumin; PSA, porcine serum albumin; RSA, rat serum albumin).

The *K_SV_* and *K* values of mycotoxin–albumin complexes were determined using the Stern–Volmer equation (Equation (1)) and the HyperQuad2006 software [11] (see data in Table 1 and Table 2, respectively). The Stern–Volmer plots (Figure 5) showed acceptable linearity (R^2^ = 0.920 to 0.996) with the 1:1 stoichiometry model. The HyperQuad analyses also supported these results. The log*K_SV_* and log*K* values of the mycotoxin–albumin complexes were in good agreement (Table 1 and Table 2).

Based on these data, AG and AMG form similarly stable complexes with these proteins (*K* ≈ 3 × 10^4^ L/mol), suggesting moderate interactions with albumins, without relevant species-dependent variations. As has been reported previously, alternariol formed more stable complexes with albumins (4 × 10^5^ L/mol to 3 × 10^6^ L/mol) [10] than its glucoside metabolites tested in the present study. In addition, RSA bound alternariol with almost eightfold higher affinity compared to HSA [10]. On the other hand, similar to glucoside metabolites (Table 2), methyl and sulfate derivatives of alternariol showed minor species differences with regard to their albumin binding [16].

Since Z14Glr only slightly influenced the emission signals of BSA, PSA, and HSA, we could not determine log*K* values from the quenching studies. However, the binding constant calculated for Z14Glr-RSA (*K* ≈ 5 × 10^4^ L/mol) refers to its moderate interaction with rat albumin, which is likely higher compared to the other examined Z14Glr–albumin complexes.

### 3.2. Interaction of AG, AMG, and Z14Glr with Serum Albumins Based on Ultracentrifugation Studies

To confirm the binding constants determined in the quenching experiments, and to get insight into the interaction of Z14Glr with HSA, ultracentrifugation studies were also performed. In these investigations, albumins (with the ligands bound) were sedimented, and then the concentrations of the unbound mycotoxins were quantified from the supernatants [32].

At pH 7.4, slightly lower binding constants of AG–HSA (log*K* = 4.2) and AMG–HSA (log*K* = 4.1) complexes were calculated than in the spectroscopic experiments; nevertheless, ultracentrifugation verified that both AG and AMG form moderately strong complexes with the protein. Furthermore, at physiological pH, the log*K* value of the Z14Glr–HSA complex was 4.2, showing the moderate interaction of this mycotoxin metabolite with human albumin.

Since spectroscopic studies suggested the higher affinity of Z14Glr toward RSA (compared to the other albumins tested), Z14Glr–RSA interaction was also examined with ultracentrifugation in PBS (pH 7.4). Based on these experiments, the log*K* value of the Z14Glr–RSA complex was 4.87 (±0.08), demonstrating that RSA binds Z14Glr 4.5-fold stronger than HSA. This is consistent with our previous observations that zearalenone and its metabolites typically form more stable complexes with RSA compared to other albumins tested [11].

Thereafter, the impacts of environmental pH on mycotoxin–HSA interactions were tested. In aqueous solutions, the extended (E) form (<pH 2.7), the fast-migrating (F) form (pH 2.7 to 4.3; loss of α-helix), the normal (N) form (pH 4.3 to 8.0; typical “heart-shaped” structure), or the basic (B) form (>pH 8.0; loss of α -helix, and sometimes increased ligand-binding affinity) of HSA occur [9]. Therefore, ultracentrifugation experiments were also carried out with HSA in sodium acetate (pH 5.0) and sodium borate (pH 8.5) buffers. At pH 5.0, pH 7.4, and pH 8.5, we noticed only minor differences in the binding constants of AG–HSA complexes (Table 3). However, AMG–HSA interaction was strongly influenced by the pH, where AMG bound to the protein with approximately 17-fold and 9-fold higher affinity at pH 8.5 than at pH 5.0 and at pH 7.4, respectively (Table 3). In accordance with this observation, alkaline conditions also strongly favored the complex formation of alternariol-9-monomethylether with albumin [16]. Furthermore, Z14Glr–HSA interaction showed only slight pH dependence (Table 3). 

### 3.3. Interaction of AG, AMG, and Z14Glr with Sulfobutylether-β-Cyclodextrin and Sugammadex

Based on previous studies, alternariol and some of its derivatives form relatively stable complexes with SBECD and SGD [16,18]. Therefore, we selected these CDs to test their complex formation with AG, AMG, and Z14Glr. Water molecules typically decrease the fluorescence signals of aromatic fluorophores [34]. Furthermore, the accommodation of a ligand in the CD cavity partially disrupts the hydration shell of the guest molecule [19]. Considering these principles, the lower quenching impacts of water molecules lead to a higher emission signal of the fluorophore [18].

**Figure 6:**
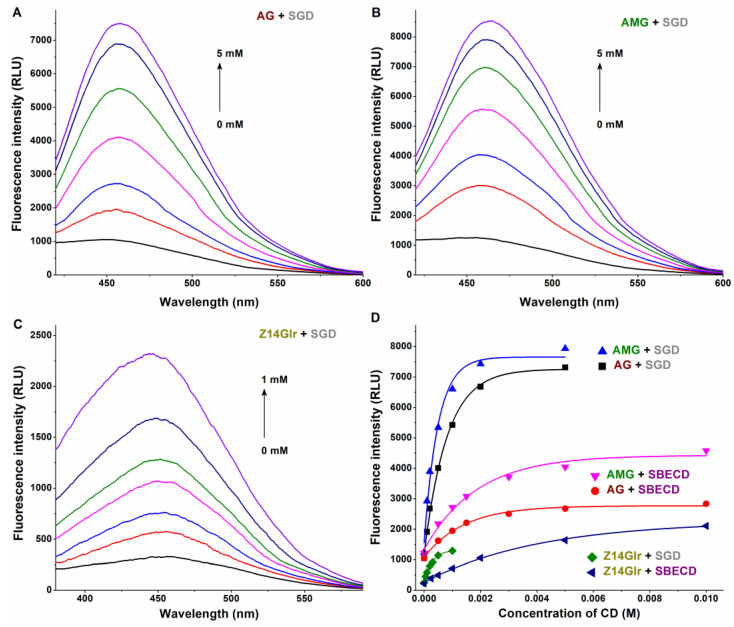
Representative fluorescence emission spectra of AG-SGD (**A**), AMG-SGD (**B**), and Z14Glr-SGD (**C**) complexes. Effects of SBECD and SGD on the fluorescence emission signals (**D**) of AG, AMG, and Z14Glr in sodium acetate buffer (0.05 M, pH 5.0; mycotoxins concentration: 1 μM; *n* = 3; λ_ex_ = 335 nm for AG and AMG, and 315 nm for Z14Glr; λ_em_ = 455 nm for each mycotoxin; AG, alternariol-3-glucoside; AMG, alternariol-9-monomethylether-3-glucoside; Z14Glr, zearalenone-14-glucuronide; SBECD, sulfobutylether-β-cyclodextrin; SGD, sugammadex).

Therefore, CD-induced elevation in the fluorescence emission intensities of mycotoxins was examined. SGD caused an approximately sevenfold increase in the emission signals of AG and AMG, while SBECD led to only threefold elevation in the fluorescence intensity of these mycotoxins (Figure 6). Furthermore, in the presence of SBECD and SGD, ninefold and fivefold increases were observed in the emission signal of Z14Glr, respectively (Figure 6). 

**Figure 7:**
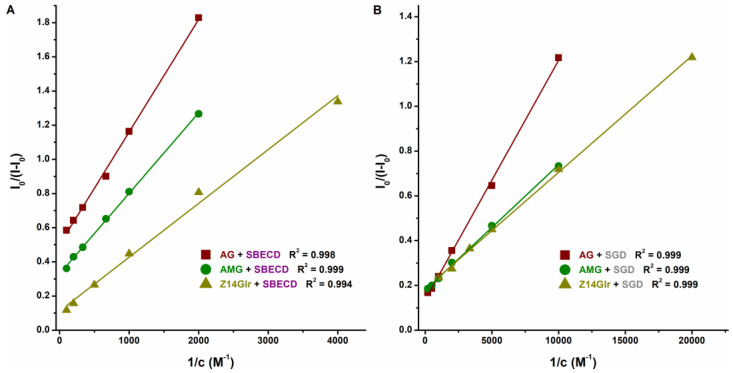
Benesi–Hildebrand plots of mycotoxin–SBECD (**A**) and mycotoxin–SGD (**B**) complexes (0.05 M sodium acetate buffer, pH 5.0; mycotoxins concentration: 1 μM; *n* = 3; λ_ex_ = 335 nm for AG and AMG, and 315 nm for Z14Glr; λ_em_ = 455 nm for each mycotoxin; AG, alternariol-3-glucoside; AMG, alternariol-9-monomethylether-3-glucoside; Z14Glr, zearalenone-14-glucuronide; SBECD, sulfobutylether-β-cyclodextrin; SGD, sugammadex).

Based on the emission signals at 455 nm, the binding constants of ligand–CD complexes were calculated using the Benesi–Hildebrand equation (Equation (2)). Background corrections were performed in each experiment. The Benesi–Hildebrand plots showed excellent fitting (R^2^ = 0.994 to 0.999) with the 1:1 stoichiometry model (Figure 7). SGD formed more stable complexes with AG, AMG, and Z14Glr compared to SBECD (Table 4). Nevertheless, the mycotoxin–SGD complexes examined here showed only moderate stability (*K* ≈ 10^3^ L/mol). These results suggest considerably weaker interactions of AG, AMG, and Z14Glr with CDs compared to the parent mycotoxins alternariol [18] and zearalenone [20]. Glucose and glucuronic acid are bulky, hydrophilic molecules; therefore, it is reasonable to hypothesize that these substituents make the inclusion of AG, AMG, and Z14Glr by the apolar CD cavity difficult.

### 3.4. Extraction of Mycotoxins from Aqueous Solutions with Cyclodextrin Bead Polymers

We did not observe the formation of highly stable complexes of AG, AMG, and Z14Glr with the tested CDs (Table 4). Nevertheless, the complex structural network of CD polymers can result in unexpectedly high ligand binding, due to the phenomenon known as cooperativity [27]. Therefore, the interactions of AG, AMG, and Z14Glr with insoluble β- (BBP) and γ-CD bead polymers (GBP) were also investigated.

**Figure 8:**
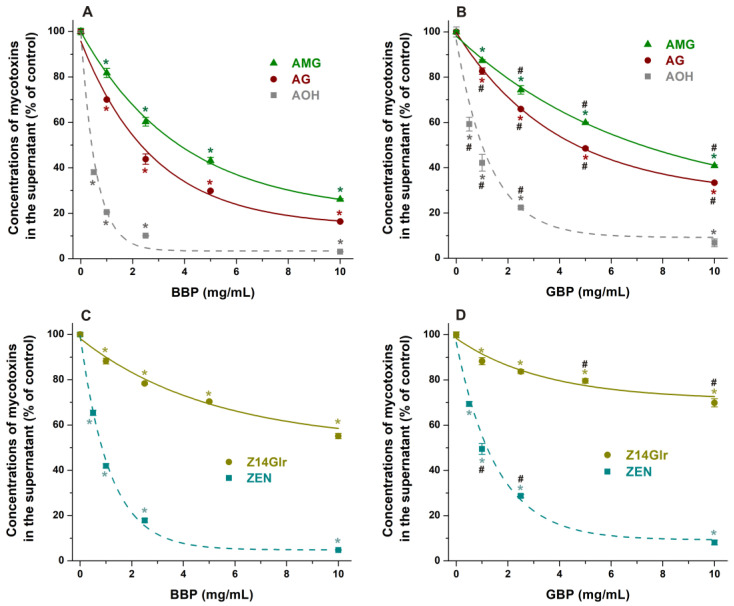
Impacts of insoluble β-CD bead polymer (BBP; (**A**,**C**)) and γ-CD bead polymer (GBP; (**B**,**D**)) on mycotoxin levels in sodium acetate buffer (0.05 M, pH 5.0), where alternariol-3-glucoside (AG), alternariol-9-monomethylether-3-glucoside (AMG), and zearalenone-14-glucuronide (Z14Glr) solutions (each 5 μM) were treated with increasing amounts of these polymers (0–10 mg/mL; see further experimental details in Section 2.4). For comparison, the extraction of the parent mycotoxins alternariol (AOH) and zearalenone (ZEN) by BBP and GBP is also presented (marked with dashed lines). Data represent the mean and standard error of the mean (SEM) (*n* = 3). Statistical significance was established based on one-way ANOVA with Tukey’s post hoc test (* *p* < 0.01: significant difference compared to the control; ^#^ *p* < 0.01: significant difference between BBP and GBP, see in panels (**B**,**D**)).

Both BBP and GBP strongly reduced the levels of AG and AMG in sodium acetate buffer (pH 5.0), where the β-derivative proved to be the more successful binder of these mycotoxins (Figure 8A,B). BBP (10 mg/mL) resulted in approximately 85% and 75% decreases in the concentrations of AG and AMG, respectively. Nevertheless, the CD-induced removal of glucoside metabolites was lower compared to the extraction of the parent mycotoxin alternariol (Figure 8A,B).

In sodium acetate buffer (pH 5.0), BBP caused a larger decrease in Z14Glr content than GBP; however, both polymers induced only moderate removal of Z14Glr compared to the parent mycotoxin zearalenone (Figure 8C,D). BBP (10 mg/mL) extracted only 45% of Z14Glr, while the same amount of this polymer removed more than 95% of zearalenone. 

**Figure 9:**
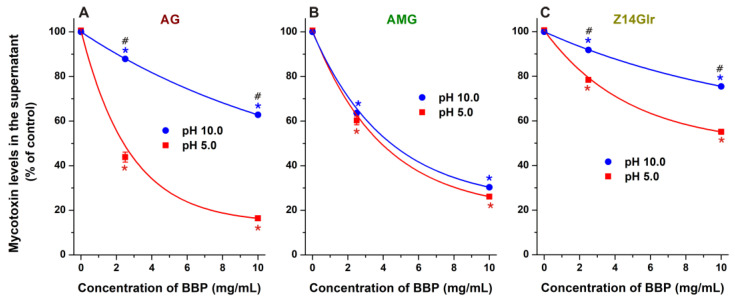
Extraction of alternariol-3-glucoside (AG; (**A**)), alternariol-9-monomethylether-3-glucoside (AMG; (**B**)), and zearalenone-14-glucuronide (Z14Glr; (**C**)) by insoluble β-CD bead polymer (BBP) from sodium acetate (pH 5.0) and sodium borate (pH 10.0) buffers (mycotoxins concentration: 5 μM). Data represent the mean and standard error of the mean (SEM) (*n* = 3). Statistical significance was established based on one-way ANOVA with Tukey’s post hoc test (* *p* < 0.01: significant difference compared to the control; ^#^ *p* < 0.01: significant difference between the toxin removal at pH 5.0 and pH 10.0).

Finally, we tested the extraction of AG, AMG, and Z14Glr by BBP under alkaline conditions (pH 10.0). Under these circumstances, the removal of AG (Figure 9A) and Z14Glr (Figure 9C) was significantly lower than at acidic pH. However, the extraction of AMG did not show relevant pH dependence (Figure 9B). The deprotonation of the phenolic hydroxyl groups of mycotoxins typically decreases their binding affinity toward uncharged CDs, likely due to the formation of less stable complexes with the anions formed [19]. The minor impact of the pH on AMG extraction can be explained by the substitution of both 3- and 9-hydroxyl groups of alternariol, while the only phenolic hydroxyl group left loses its proton only at higher pH, and/or the dissociation of this proton has no large impact on the AMG–CD complex formation.

## 4. Conclusions

In summary, this is the first investigation to examine the interactions of AG, AMG, and Z14Glr with serum albumins and CDs. AG and AMG formed moderately strong complexes with the tested albumins, without relevant species variations. However, Z14Glr showed 4.5-fold stronger interaction with RSA than with HSA. Furthermore, alkaline conditions favored the interaction of AMG with HSA, resulting in ninefold higher stability of AMG–HSA complexes at pH 8.5 than at pH 7.4. Mycotoxin–CD complexes showed weak-to-moderate stability, where SGD formed more stable complexes with AG, AMG, and Z14Glr than SBECD did. BBP was a more effective binder of AG, AMG, and Z14Glr compared to GBP. CD bead polymers caused moderate removal of AG and AMG from aqueous solutions, while less effective extraction of Z14Glr was observed. At pH 10.0, BBP removed considerably lower amounts of AG and Z14Glr than at pH 5.0, while the extraction of AMG did not show relevant pH dependence. These observations demonstrate that albumins and CDs bind AG, AMG, and Z14Glr with lower affinity compared to the parent mycotoxins alternariol and zearalenone. Nevertheless, our novel findings demonstrate that RSA can be considered as a relatively strong binder of Z14Glr, and the stability of AMG–HSA complexes was high at pH 8.5. Therefore, albumins can be considered as affinity proteins with regard to the latter mycotoxin metabolites. As another important observation, BBP removed approximately 85% and 75% of AG and AMG, respectively. Therefore, CD polymers could be important in the development of CD-based toxin extraction strategies (e.g., removal of mycotoxins from certain beverages) with regard to both alternariol and its masked derivatives.

## Figures and Tables

**Table 1:** Decimal logarithmic values of Stern–Volmer quenching constants (*K_SV_*; L/mol) of mycotoxin–albumin complexes, determined based on fluorescence quenching experiments (*n* = 3).

	HSA log*K_SV_* ± SEM	BSA log*K_SV_* ± SEM	PSA log*K_SV_* ± SEM	RSA log*K_SV_* ± SEM
**AG**	4.48 ± 0.08	4.44 ± 0.04	4.38 ± 0.03	4.49 ± 0.03
**AMG**	4.38 ± 0.05	4.57 ± 0.02	4.27 ± 0.03	4.62 ± 0.02
**Z14Glr**	–	–	–	4.65 ± 0.05

AG, alternariol-3-glucoside; AMG, alternariol-9-monomethylether-3-glucoside; Z14Glr, zeara-lenone-14-glucuronide; HSA, human serum albumin; BSA, bovine serum albumin; PSA, porcine serum albumin; RSA, rat serum albumin; SEM, standard error of the mean.

**Table 2:** Decimal logarithmic values of binding constants (*K*; L/mol) of mycotoxin–albumin complexes, determined based on fluorescence quenching experiments (*n* = 3).

	HSA log*K* ± SEM	BSA log*K* ± SEM	PSA log*K* ± SEM	RSA log*K* ± SEM
**AG**	4.52 ± 0.10	4.46 ± 0.04	4.43 ± 0.03	4.53 ± 0.02
**AMG**	4.40 ± 0.05	4.59 ± 0.02	4.29 ± 0.02	4.65 ± 0.02
**Z14Glr**	–	–	–	4.71 ± 0.03

AG, alternariol-3-glucoside; AMG, alternariol-9-monomethylether-3-glucoside; Z14Glr, zeara-lenone-14-glucuronide; HSA, human serum albumin; BSA, bovine serum albumin; PSA, porcine serum albumin; RSA, rat serum albumin; SEM, standard error of the mean.

**Table 3:** Decimal logarithmic values of binding constants (*K*; L/mol) of mycotoxin–albumin complexes, determined based on ultracentrifugation experiments (*n* = 3).

	HSA pH 7.4 log*K* ± SEM	HSA pH 5.0 log*K* ± SEM	HSA pH 8.5 log*K* ± SEM
**AG**	4.24 ± 0.02	4.48 ± 0.03	4.40 ± 0.06
**AMG**	4.11 ± 0.03	3.82 ± 0.02	5.05 ± 0.07
**Z14Glr**	4.22 ± 0.03	4.59 ± 0.14	4.23 ± 0.16

AG, alternariol-3-glucoside; AMG, alternariol-9-monomethylether-3-glucoside; Z14Glr, zeara-lenone-14-glucuronide; SEM, standard error of the mean.

**Table 4:** Decimal logarithmic values of the binding constants (*K*; L/mol) of mycotoxin–cyclodextrin complexes, determined based on fluorescence spectroscopic studies (*n* = 3).

	SBECD logK ± SEM	SGD logK ± SEM
**AG**	2.92 ± 0.05	3.19 ± 0.08
**AMG**	2.79 ± 0.08	3.45 ± 0.04
**Z14Glr**	2.32 ± 0.12	3.46 ± 0.05

AG, alternariol-3-glucoside; AMG, alternariol-9-monomethylether-3-glucoside; Z14Glr, zeara-lenone-14-glucuronide; SBECD, sulfobutylether-β-cyclodextrin; SGD, sugammadex; SEM, standard error of the mean.

## Data Availability

Data will be made available upon request due to privacy or ethical restrictions.

## Supplementary Materials

The following supporting information can be downloaded at: https://www.mdpi.com/article/10.3390/metabo13030446/s1, Figure S1: Synthesis of zearalenone-14-β,D-glucuronide. [35,36]
