# Supplementary material for: Probing Serum Albumins and Cyclodextrins as Binders of the Mycotoxin Metabolites Alternariol-3-Glucoside, Alternariol-9-Monomethylether-3-Glucoside, and Zearalenone-14-Glucuronide
Source: Metabolites. 2023 Mar 18;13(3):446. doi: 10.3390/metabo13030446 (PMC10059066; doi:10.3390/metabo13030446)
Supplement: Supplementary file 1 [file metabolites-13-00446-s001.zip › metabolites-2259193-supplementary.pdf]

Probing Serum Albumins and Cyclodextrins as Binders of the Mycotoxin Metabolites Alternariol-3-Glucoside, Alternariol-9-Monomethylether-3-Glucoside, and Zearalenone-14-Glucuronide

SUPPLEMENTARY MATERIALS

Miklós Poór [1,2,*], Beáta Lemli [1,3], Péter Vilmányi [1], Ágnes Dombi [1], Zoltán Nagymihály [4], Eszter Borbála Both [4], Nándor Lambert [4], Tamás Czömpöly [2,4] and Lajos Szente [5]

[1] Department of Pharmacology, Faculty of Pharmacy, University of Pécs, Rókus u. 2, H-7624 Pécs, Hungary
[2] Food Biotechnology Research Group, János Szentágothai Research Centre, University of Pécs, Ifjúság útja 20, H-7624 Pécs, Hungary
[3] Green Chemistry Research Group, János Szentágothai Research Centre, University of Pécs, Ifjúság útja 20, H-7624 Pécs, Hungary
[4] Soft Flow Ltd., Pellérdi út 91/B, H-7634 Pécs, Hungary
[5] CycloLab Cyclodextrin Research & Development Laboratory, Ltd., H-1097 Budapest, Hungary
***** Correspondence: poor.miklos@pte.hu (M.P.)

Synthesis of zearalenone-14-β,D-glucuronide (Z14Glr):

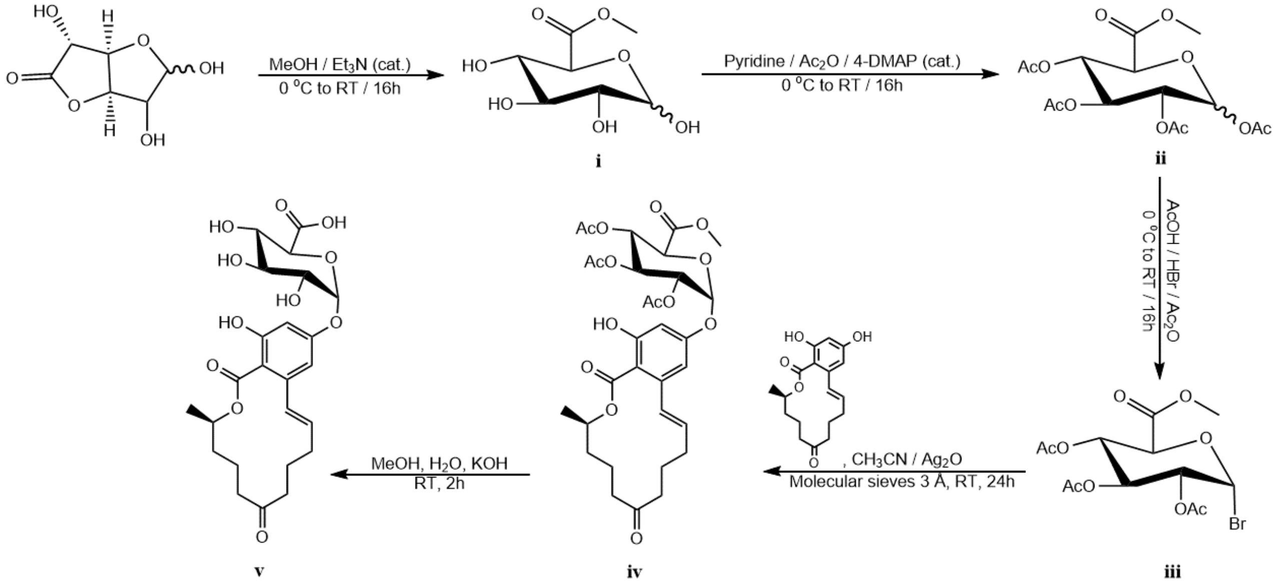

Figure S1. Synthesis of zearalenone-14-β,D-glucuronide.

The synthesis of zearalenone-14-β,D-glucuronide was carried out as described by Mikula et al. (2012) [1] and Nasseri et al. (2018) [2], with the modifications of the following steps:

*Synthesis of 1,2,3,4-tetra-O-acetyl-β,D-glucuronic acid methyl ester (**ii**):*

Continuous stirring was applied throughout the whole procedure to achieve sufficient mixing of the reaction mixture. First, 50 µL (~ 0.02 eq) of triethylamine was added to 30 mL of methanol kept at 0 °C in an ice-water bath. After 3 min of mixing of the methanol/triethylamine solution, 3.00 g of D-(+)-glucuronic acid γ-lactone was added step by step (500 mg doses every 4 minutes) to the reaction mixture. The reactor vapor space was sealed by flushing with argon through a reflux condenser and further stirred at 0 °C. After 80 min, a further 50 µL of triethylamine was added to the reaction mixture and placed at room temperature for 16 h. The reaction produced a yellow homogeneous solution, which was dried under nitrogen, creating a deep yellow oil (**i**), and passed to the next esterification reaction step. The yellowish oil-like substance (**i**) was dissolved in 30 mL of pyridine and placed in an ice bath while stirring continuously. After adding 100 mg of 4-

dimethylaminopyridine (4-DMAP) to the reaction mixture, 14 mL of acetic anhydride was added dropwise to the solution slowly over the course of 30 min while constantly mixing. When one-third of the acetylating agent was added, the solution became clear and dark in color. This color became progressively darker as the acetic anhydride was introduced to the reaction mixture, until it turned deep black. The solution was held at room temperature for overnight reaction, filtered through Celite, and dried under a nitrogen flow. The crude residue was dissolved again in dichloromethane (50 mL) and washed twice with water (2 x 50 mL). The organic layer was dried on $MgSO_4$ and evaporated until dry. Product (**ii**) was obtained as white crystals (5.32 g, yield: 83%). The synthesized compound was confirmed by ESI-MS analysis, showing m/z = 399.19 $[M+Na]^+$ [2].

*Synthesis of 2,3,4-tri-O-acetyl-1-bromo-1-deoxy-α,D-glucopyranuronic acid methyl ester (**iii**):*

The reaction was performed under an argon atmosphere in a round-bottomed flask by using a reflux condenser and argon ball on the top, while constantly mixing with a magnetic stirring ball. First, 1.46 g (3.88 mmol) of 1,2,3,4-tetra-*O*-acetyl-β,D-glucuronic acid methyl ester (**ii**) and 23.4 mL of hydrogen bromide in acetic acid solution (HBr 33 wt. % in acetic acid) were measured in the round-bottomed flask and mixed with 3.2 mL of acetic anhydride solution. The reaction mixture was kept at room temperature, avoiding light, and mixed for 16 h, before being filtered through Celite and evaporated until dry. The crude residue was dissolved again in dichloromethane (50 mL) and washed twice with water (2 x 50 mL). The organic layer was dried on anhydrous $MgSO_4$ and after filtration it was evaporated under a nitrogen flow. Product (**iii**) was obtained as white crystals (1.14 g, yield: 74%). The synthesized compound was confirmed by ESI-MS analysis, showing m/z = 419.14 and 421.24 $[M+Na]^+$ isotopologues.

*Synthesis of zearalenone-14-(2,3,4-tri-O-acetyl-β,D-glucuronic acid methyl ester) (**iv**):*

The reaction was performed under an argon atmosphere in a round-bottomed flask by using a reflux condenser and argon ball on the top, while constantly mixing with a magnetic stirrer. The flask was flushed thoroughly with argon before adding the reagents, and 100 mg of powdered molecular sieves (3Å) was added. Then, 31.8 mg (0.1 mmol) of zearalenone and 99.3 mg (0.25 mmol) of 2,3,4-tri-*O*-acetyl-1-bromo-1-deoxy-α,D-glucopyranuronic acid methyl ester (**iii**) were

dissolved in 4 mL of anhydrous acetonitrile in the prepared round-bottomed flask. The reaction mixture was stirred in the dark for 3 h at room temperature. The stirring was continued for 24 h in the dark after the addition of 34.8 mg (0.15 mmol) of Ag_2O as an activator. Then, 34.8 mg (0.15 mmol) of Ag_2O and 99.3 mg (0.25 mmol) of 2,3,4-tri-*O*-acetyl-1-bromo-1-deoxy-α-D-glucopyranuronic acid methyl ester (**iii**) were added. The conversion of the reaction was monitored by HPLC, and the reaction was stopped after 30 h. The reaction mixture was filtered through Celite and evaporated until dry. The crude residue was dissolved again in dichloromethane (50 mL) and washed twice with water (2 x 50 mL). The organic layer was dried on $MgSO_4$ and evaporated until dry. The resulting residue was purified by preparative HPLC to obtain pure zearalenone-14-(2,3,4-tri-*O*-acetyl-β,D-glucuronic acid methyl ester). Product (**iv**) was obtained as a white powder (44.2 mg, yield: 69%). The HPLC purification was carried out on a Knauer preparative system coupled with a Waters QDa mass spectrometer using a Phenomenex Axia C18 (20 mm×250 mm) column and a mixture of acetonitrile and water as the mobile phase. The corresponding compound was confirmed by m/z = 657.39 $[M+Na]^+$.

*Synthesis of zearalenone-14-β,D-glucuronide (**v**):*

First, 44.2 mg (0.07 mmol) of zearalenone-14-(2,3,4-tri-*O*-acetyl-β,D-glucuronic acid methyl ester) (**iv**) was dissolved in 5 mL of tetrahydrofuran in a 25 mL round-bottomed flask; then, 0.5 mL of Claisen's alkali (prepared by dissolving 350 g of KOH in 250 mL of water, cooling, and diluting to 1000 mL with methanol) was added to the reaction mixture. The reaction mixture was heated to 40 °C for 4 h with continuous stirring (until full conversion was indicated by the HPLC measurements). When the reaction was completed, the mixture was cooled to room temperature and the solution was acidified with 2 M hydrochloric acid. The resulting mixture was poured into 10 mL of ethyl acetate and washed twice with water (2 x 20 mL). The organic layer was dried on $MgSO_4$ and evaporated until completely dry. The resulting residue was purified by preparative HPLC, as described above, to obtain zearalenone-14-β,D-glucuronide (**v**) with a yield of 25.1 mg (73%). The successful synthesis and purification of zearalenone-14-β,D-glucuronide (**v**) was confirmed with ESI-MS analysis, resulting in m/z = 517.25 $[M+Na]^+$, and with the following ^{1}H- and ^{13}C-NMR measurements:

^{1}H-NMR (500 MHz, DMSO-d6): δ 1.26 (d, J = 6.2 Hz), 1.51 (m, 2H), 1.65 (m, 2H), 1.78 (2H), 2.03 (1H), 2.18 (1H), 2.20 (1H), 2.30 (m), 2.37 (m), 2.64 (m, 1H), 3.25 (ddd, J = 2.7, 11.2, 18.7 Hz), 3.87 (d, J = 9.8 Hz), 5.06 (d, J = 7.7 Hz), 5.24 (m, 1H), 5.43 (m, 1H), 6.02 (ddd, J = 4.7, 9.6, 15.4 Hz), 6.39 (d, J = 2.6 Hz), 6.46 (s, 1H), 6.65 (s, 1H), 8.15 (s, 1H), 10.52 (s, 1H).

^{13}C-NMR (125 MHz, DMSO-d6): δ 20.3, 21.5, 21.5, 31.4, 34.8, 37.1, 43.5, 71.9, 72.0, 73.4, 75.7, 76.3, 100.3, 103.1, 105.3, 112.9, 129.9, 133.5, 138.6, 158.6, 159.7, 168.7, 170.6, 211.1.

References:

[1] Mikula, H.; Hametner, C.; Berthiller, F.; Warth, B.; Krska, R.; Adam, G.; Fröhlich, J. Fast Reproducible Chemical Synthesis of Zearalenone-14-β,D-Glucuronide. *World Mycotoxin Journal*, **2012**, 5, 289–296. https://doi.org/10.3920/wmj2012.1404.

[2] Nasseri, S.A.; Betschart, L.; Opaleva, D.; Rahfeld, P.; Withers, S.G. A Mechanism-Based Approach to Screening Metagenomic Libraries for Discovery of Unconventional Glycosidases. *Angewandte Chemie International Edition*, **2018**, 57, 11359–11364. https://doi.org/10.1002/anie.201806792.
